# 2bRAD-M reveals differences in microbial communities between Modic changes and disc herniation

**DOI:** 10.3389/fcimb.2025.1449873

**Published:** 2025-03-21

**Authors:** Fang Yang, Jingtao Zhang, Junpu Zha, Guolei Zhang, Jia Li, Wei Du, Lin Liu, Jun Di

**Affiliations:** ^1^ Department of Radiotherapy, The Fourth Hospital of Hebei Medical University, Shijiazhuang, Hebei, China; ^2^ Department of Orthopedics, The Third Hospital of Hebei Medical University, Shijiazhuang, Hebei, China

**Keywords:** Modic change, disc herniation, lumbar spine, microbiome, 2bRAD-M, bacteria

## Abstract

**Background:**

Modic changes are caused by various factors, such as degenerative processes, inflammation, biomechanical, genetic, and metabolic factors, infection, and smoking. Bacteria have been identified in human intervertebral discs by 16S rRNA sequencing; however, the low microbial biomass in intervertebral disc tissue limits species-level analyses using this approach. In this study, we employed 2bRAD-M (2b Restriction Site Associated DNA sequencing for Microbiome), a new sequencing technology capable of accurately characterizing bacteria, fungi, and archaea in samples with low microbial biomass at species-level resolution.

**Methods:**

We surveyed 20 intervertebral disc (IVD) samples, including 10 IVD samples with Modic changes and 10 herniated disc samples. 2bRAD-M was performed to explore whether microbial differences existed between Modic change and herniated disc samples.

**Results:**

In total, 332 microbial species were identified, including 75 species shared between the two groups. Enrichment for *Escherichia_coli*, *Cupriavidus_pauculus*, and *Bradyrhizobium_denitrificans* was observed in the Modic change group, while *Afipia_broomeae*, *Phyllobacterium_calauticae*, *Tardiphaga_sp002256345*, *Mesorhizobium_sp004136315*, *Afipia_sp000497575*, *Burkholderia_contaminans*, and *Afipia_sp017474385* were more abundant in the herniated disc group. Additionally, 19 discriminatory taxa were determined by linear discriminant analysis effect size (LEfSe). In a random forest model for partitioning the two groups, the species with the highest variable importance were *Afipia_broomeae*, *Phyllobacterium_calauticae*, and *Escherichia_coli*. Moreover, a newly constructed random forest model based on an optimal marker set consisting of eight highly abundant species successfully distinguished between the Modic change and herniated disc groups, with an accuracy of 81.0%. A functional annotation analysis showed that differentially abundant taxa between the Modic change and herniated disc groups could be assigned to 4093 COGs (Clusters of Orthologous Groups) and 342 related signaling pathways.

**Conclusion:**

This study represents the first application of 2bRAD-M to Modic changes and disc herniation, revealing significant differences in microbial taxa between the two groups. These results suggest that microbial dysbiosis in the intervertebral disc is associated with Modic changes and provide candidate targets for further studies of the mechanisms underlying the development and progression of Modic changes.

## 1 Introduction

The global prevalence of low back pain is high, affecting approximately 700 million individuals worldwide, with similar frequencies across various regions and populations (James et al., 2018; Jin et al., 2020). It is one of the most common reasons for seeking medical attention and can have significant impacts on quality of life and productivity. Our understanding of low back pain and degenerative disc disease has expanded beyond mechanical and genetic factors. For example, there is some evidence that microbial factors, including intervertebral disc (IVD) microbes, contribute to these conditions (Rajasekaran et al., 2020a; Shanmuganathan et al., 2022; Rajasekaran et al., 2023b). This evolving understanding underscores the complexity of the human microbiome and its impact on health as well as the complex interplay between the gut, immune system, and nervous system. In particular, the gut microbiota can influence systemic inflammation and pain sensitivity, potentially contributing to low back pain and fibromyalgia (Minerbi et al., 2019; Shmagel et al., 2019). Ongoing research is focused on the role of microbes, particularly those found in IVDs with Modic changes on MRI indicative of a distinct phenotype characterized by heightened pain intensity, frequent episodes of low back pain, limited response to conservative therapy, and an increased likelihood of surgical intervention (Kjaer et al., 2006; Zhang et al., 2008). Modic changes, particularly Type I, are associated with inflammation and infection. Understanding the role of the microbiome could provide new directions for the treatment and prevention of low back pain (Chen et al., 2016; Granville Smith et al., 2022; Dhariwal et al., 2023).

Even asymptomatic individuals have a distinct microbiome within their lumbar IVDs, suggesting that microbial colonization may be a normal aspect of disc health. This challenges traditional views of the spine as a sterile environment and opens up new avenues for exploring the role of microbes in spinal health and disease. Comparisons of microbial diversity between patients with herniated discs and Modic changes is a promising approach for identifying diagnostic biomarkers and specific microbial signatures associated with different types of disc pathology. These biomarkers could aid in more accurate diagnosis and possibly lead to personalized treatment strategies for individuals with IVD diseases.

Studies of the IVD microbiome have used 16S rRNA sequencing. However, owing to its limited sensitivity and resolution, this method generally enables genus-level classification, and the identification of microbes at the species level is difficult. 2bRAD-M is a novel high-throughput sequencing technology designed to accurately characterize bacteria, fungi, and archaea in samples with low microbial biomass at species-level resolution. It is a cost-effective alternative to whole metagenome shotgun sequencing, which is expensive and requires a large initial biomass. 2bRAD-M provides species-level resolution, effectively identifies host contamination, and handles sample degradation, making it ideal for studying low-biomass samples such as IVD tissues (Lam et al., 2022; Sun et al., 2022).

The clinical classification of Modic changes is traditionally based on MRI, which identifies three types of changes. While MRI is a valuable tool for visualizing structural changes, it does not provide insights into the underlying biological processes, such as microbial involvement. Our study aims to complement MRI findings by analyzing the microbiota in IVDs with Modic changes and herniated discs. By identifying distinct microbial signatures, we can enhance the accuracy of diagnosis and potentially lead to personalized treatment strategies. In the present study, 10 patients with Modic changes and 10 patients with IVD herniation were evaluated using 2bRAD-M to compare the microbial compositions in the two groups. This is the first application of the 2bRAD-M approach to analyze microbial taxa associated with Modic changes at the species level.

## 2 Materials and methods

### 2.1 Sample collection

We calculated the required sample size using the following formula for a two-sample comparison of means (assuming equal variances):


$$n_{1}=n_{2}=2{\left[\frac{\left(Z_{\alpha /2}+Z_{\beta }\right)\text{σ}}{\text{δ}}\right]}^{2}$$

-
$Z_{\alpha /2}$ is the Z-value for the desired significance level (1.96 for α = 0.05).
-
$Z_{\beta }$ is the Z-value for the desired power (0.84 for power = 0.80).
-
δ is the difference in means (effect size).
- *σ* is the pooled standard deviation.

Based on the detection rate and abundance of microbial species identified in the preliminary experiment, *Escherichia_coli* was selected as the key species for this study. The required sample size was determined by evaluating the abundance difference of this key species. This study anticipates a significant difference in the abundance of *Escherichia_coli* between the two groups. According to pilot data, the standard deviation (σ) is estimated at 1.56, and the effect size (δ) at 2.0.

Sample Size Formula: $n_{1}=n_{2}=2{\left[\frac{\left(1.96+0.84\right)*1.56}{2.0}\right]}^{2}=9.5$ .

Given the practical constraints and the pilot data, we chose a sample size of 10 per group as a reasonable compromise.

**Figure 1:**
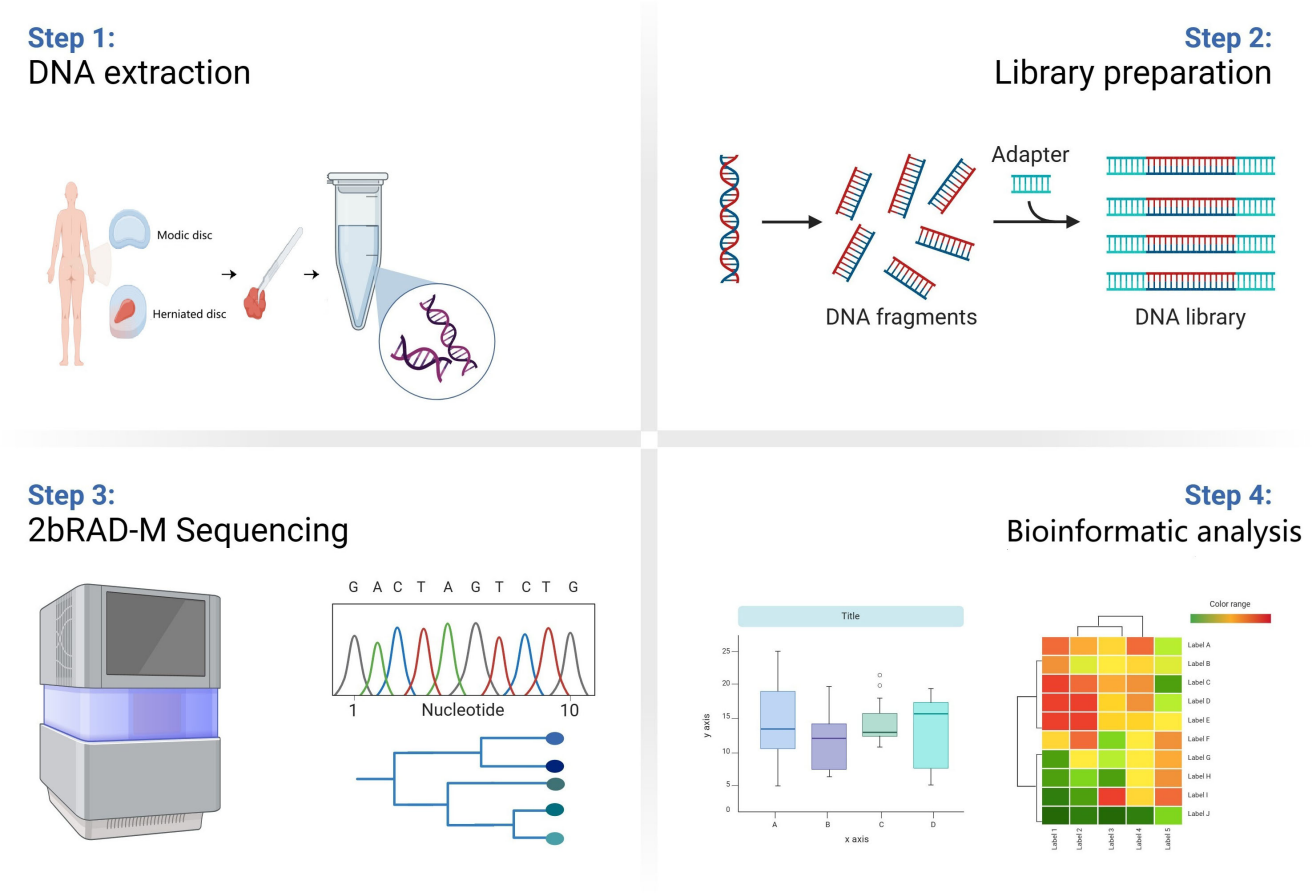
Schematic diagram of the study flow. Samples from Group M and group H patients were first collected. Then the DNA information of microbes in the samples was extracted and the microbes were compared and analyzed at the species level by 2bRAD-M technology. Finally, the data information was analyzed by multidimensional processing.

As shown in **Figure 1**, disc tissues from 10 patients with Modic changes (group M) and 10 patients with herniated discs (group H) were collected from the Third Hospital of Hebei Medical University. Modic changes were classified according to the established system (Modic et al., 1988a; Modic et al., 1988b) into three types: Type 1 (hypointense on T1 and hyperintense on T2 images), Type 2 (hyperintense on T1 and isointense/hyperintense on T2 images), and Type 3 (hypointense on both T1 and T2 images). To minimize the influence of extrinsic factors on the disc microbiome, disc tissue samples were placed in sterile freeze-storage tubes with sterile forceps and placed in liquid nitrogen without touching anything else. Inclusion criteria for patients in group M were as follows: initial diagnosis with Modic changes, followed by surgical resection. The distribution of patients in each Modic change classification is as follows: 4 patients with Type 1, 3 patients with Type 2, and 3 patients with Type 3 Modic changes. Inclusion criteria for patients in group H were as follows: initial diagnosis with lumbar disc herniation and subsequent surgical resection. The following exclusion criteria were established: (1) Participants who had undergone antibiotic therapy for any reason within two months prior to sample collection; (2) Individuals with elevated C-reactive protein (CRP) or erythrocyte sedimentation rate (ESR) levels; and/or (3) Patients exhibiting local signs of infection at the surgical site. Sample collection was approved by the Ethical Review Committee of the Third Hospital of Hebei Medical University. The samples were collected and used after obtaining informed consent from the patients. Demographic and clinical characteristics of the enrolled patients are listed in **Table 1**.

**Table 1:** Demographic and clinical characteristics of the enrolled patients (n=20).

Parameter	Number (%) or mean ± SD
Age(years)	59.6 ± 10.9
Gender	
Male	11 (55)
Female	9 (45)
Body mass index (kg/m^2^)	28.1 ± 2.8
Root pain side	
Left	5 (25)
Right	12 (60)
Both	3 (15)
Surgical level	
One-level	14 (70)
Two-level	6 (30)
Comorbidities	
Hypertension	7 (35)
Diabetes	3 (15)

### 2.2 DNA extraction, library construction, and sequencing

DNA was extracted from tissue samples using a CretMag Multi Sample DNA Kit following the manufacturer’s instructions (CretBiotech Ltd., Suzhou, China). 2bRAD-M library preparation was performed following the original protocol developed by Wang et al (Wang et al., 2012), with minor modifications. DNA (1 pg to 200 ng) was digested with 4 U of BcgI (NEB, Ipswich, MA, USA) for 3 h at 37°C. Subsequently, adaptors were ligated to the DNA fragments. The ligation reaction was performed by combining 10 µl of digested DNA with 10 µl of a ligation master mix containing 0.2 µM each of two adaptors and 800 U of T4 DNA ligase (NEB). Ligation was carried out at 4°C for 12 h. Then, ligation products were amplified, and PCR products were separated by 8% polyacrylamide gel electrophoresis. Bands of approximately 100 bp were excised from the polyacrylamide gel, and the DNA was diffused from the gel in nuclease-free water for 6–12 h at 4°C. Sample-specific barcodes were introduced by PCR with platform-specific barcode-bearing primers. Each 20 µl PCR contained 6 µl of gel-extracted PCR product, 0.2 µM each primer, 0.3 mM dNTP, 1×Phusion HF buffer, and 0.4 U of Phusion High-Fidelity DNA Polymerase (NEB). PCR products were purified using the QIAquick PCR Purification Kit (Qiagen, Hilden, Germany) and then sequenced using the Illumina Nova PE150 platform. 2bRAD-M was carried out at Qingdao OE Biotech Co., Ltd. (Qingdao, China). All adaptors and primers are listed in **Supplementary Table S1**.

### 2.3 2bRAD microbial database construction

First, 402698 microbial genomes (including bacterial and archaeal genomes) from the GTDB database and 1501 fungal genomes from the Ensembl database were obtained. Then, built-in Perl scripts were used to sample restriction fragments from microbial genomes by each of 16 type 2B restriction enzymes, forming a large 2bRAD microbial genome database. The set of 2bRAD tags sampled from each genome was assigned a GCF number as well as GCF taxonomic information corresponding to the whole genome (Sun et al., 2022; Sun JX. et al., 2023; Sun Z. et al., 2023). Finally, all 2bRAD tags from each GCF detected once within the genome were compared with those of all others. 2bRAD tags that were specific to a species-level taxon (i.e., those that had no overlap with other species) were developed as species-specific 2bRAD markers, collectively forming a 2bRAD marker database.

### 2.4 Calculation of relative abundance

To identify microbial species within each sample, all sequenced 2bRAD tags after quality control were mapped (using a built-in Perl script) against the 2bRAD marker database, containing all 2bRAD tags theoretically unique to each of 86,022 microbial species in the database. To control false-positives in species identification, a G score was derived for each species identified within a sample. The G score was defined as follows: G score _species i_ $=\sqrt{S_{i}\times t_{i}}$ (*S*: number of reads assigned to all 2bRAD markers belonging to species i within a sample; t : number of all 2bRAD markers for species *i* that have been sequenced within a sample). The threshold G score for a false positive discovery of a microbial species was set to 5 (Sun et al., 2022). The average read coverage for all 2bRAD markers was calculated for each species, representing the number of individuals belonging to a species in a sample at a given sequencing depth. The relative abundance of a given species was then calculated as follows: Relative abundance _species i_ $=\frac{S_{i}/T_{i}}{\sum_{i=1}^{n}S_{i}/T_{i}}$ (*S*: number of reads assigned to all 2bRAD markers for species *i* within a sample; *T*: number of all theoretical 2bRAD markers for species *i*).

### 2.5 Statistical analysis

R (4.2.3) software was used for statistical analyses. Indicators of alpha diversity, Chao1 (species number and richness), Shannon index (species richness and evenness) and Simpson index (species diversity), in groups M and H were compared using paired Wilcoxon tests. Beta diversity was compared between groups M and H using a principal coordinate analysis (PCoA) based on Jaccard distances for binary data, Bray–Curtis distances, and Euclidean distances. Differences in species abundance between the two groups were analyzed using the Wilcoxon test. In addition, correlations between species were evaluated using Spearman correlation analyses based on estimates of relative abundance. For functional predictions (COG and KEGG), differences between groups were analyzed by Wilcoxon tests. The results were regarded as statistically significant when P < 0.05.

## 3 Results

### 3.1 IVD microbial diversity in Modic change and herniated disc groups

**Figure 2:**
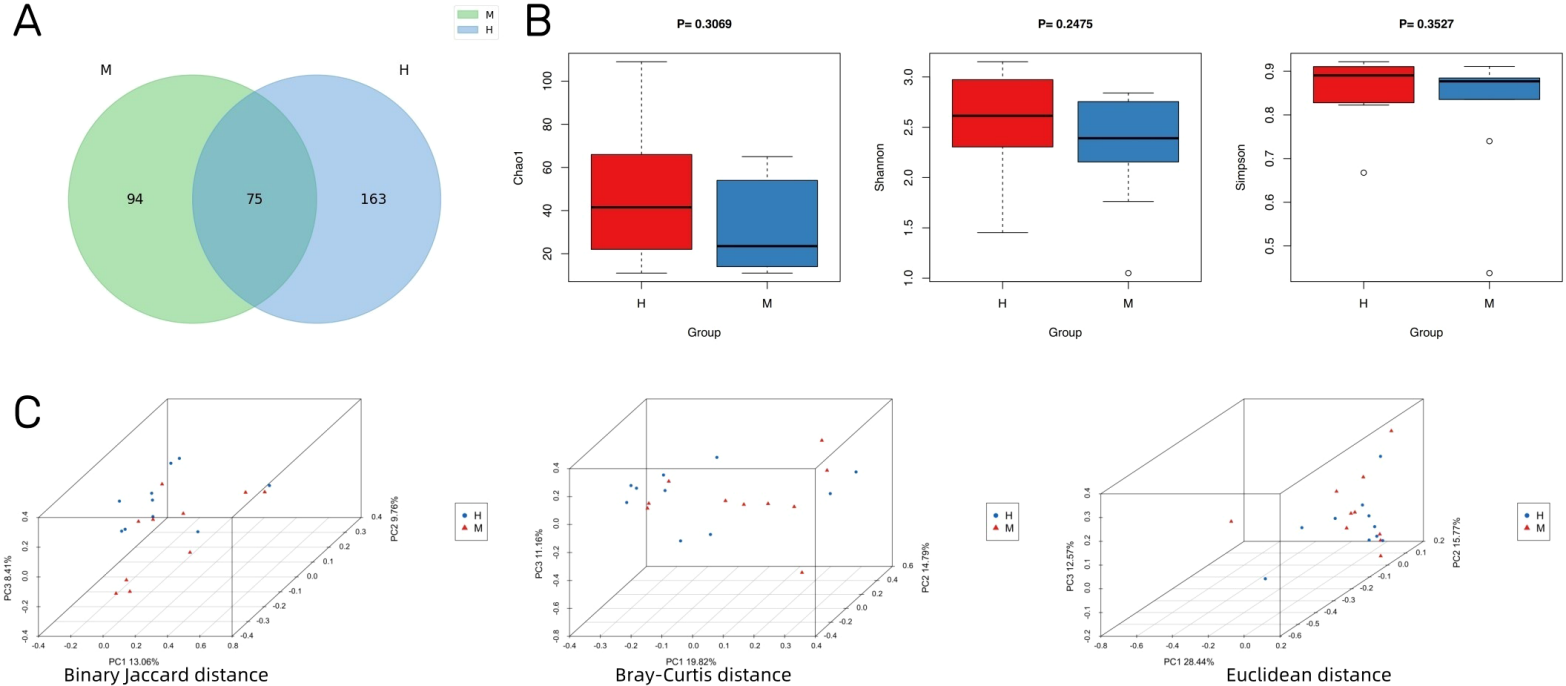
IVD microbial diversity of Modic change and herniated disc tissues. **(A)** Venn diagram showing the overlap of microbial species between Modic change and herniated disc tissues. **(B)** Comparison of differences in alpha diversity between group M and group H. **(C)** Comparison of differences in beta diversity between group M and group H based on 3D-PCoA. Each point corresponds to a sample, where the green point represents group H and the purple triangle represents group M.

An overview of the volume of sequencing data during quality control is provided in **Supplementary Table S2**, including raw reads, enzyme reads, and clean reads. A total of 332 microbial species were identified in groups M and H. As visualized using a Venn diagram, 75 species overlapped in the two groups (**Figure 2A**). Additionally, 94 species were specific to group M and 163 were specific to group H. With respect to alpha diversity, as shown in **Figure 2B**, Chao1, Shannon, and Simpson indexes did not differ significantly between group M and group H. Nevertheless, the number and diversity of species tended to be higher in group H than in group M. Additionally, we found differences in the microbial composition between groups by PCoA (**Figure 2C**).

### 3.2 IVD microbial community composition

**Figure 3:**
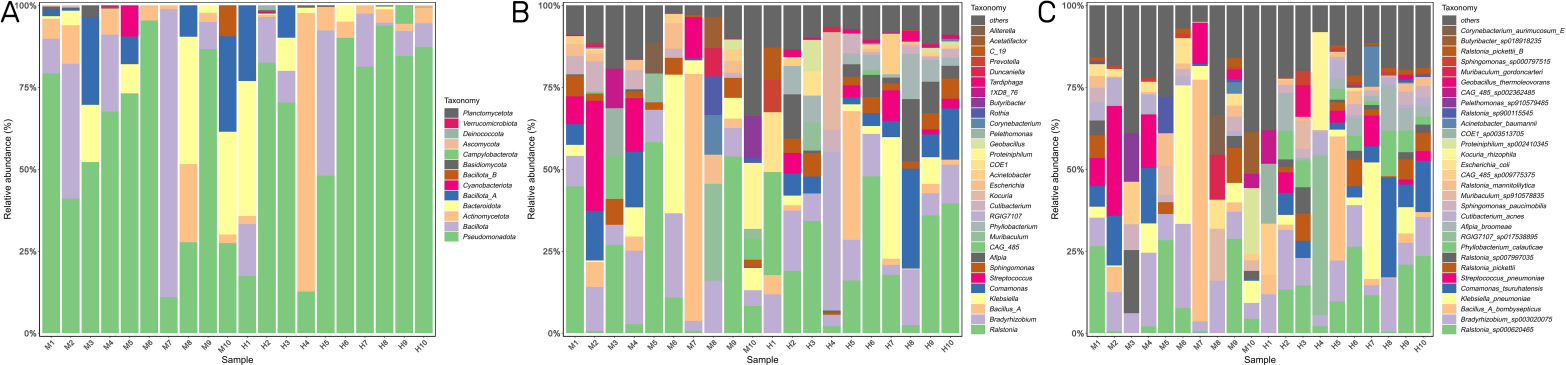
IVD microbial community composition. **(A)** The relative abundance of bacterial phylum in the two groups. **(B,C)** The top 30 most abundant bacterial genus **(B)** and species **(C)** in the two groups.

At the phylum level, Pseudomonadota, Bacillota, and Actinomycetota were the dominant phyla in the two groups (**Figure 3A**). At the genus level, *Ralstonia*, *Bradyrhizobium*, and *Bacillus_A* were dominant in both groups (**Figure 3B**). The top five most abundant species were *Ralstonia_sp000620465*, *Bradyrhizobium_sp003020075*, *Bacillus_A_bombysepticus, Klebsiella_pneumoniae*, and *Comamonas_tsuruhatensis* in both groups (**Figure 3C**).

### 3.3 Differential abundance of microbial species between Modic change and herniated disc groups

**Figure 4:**
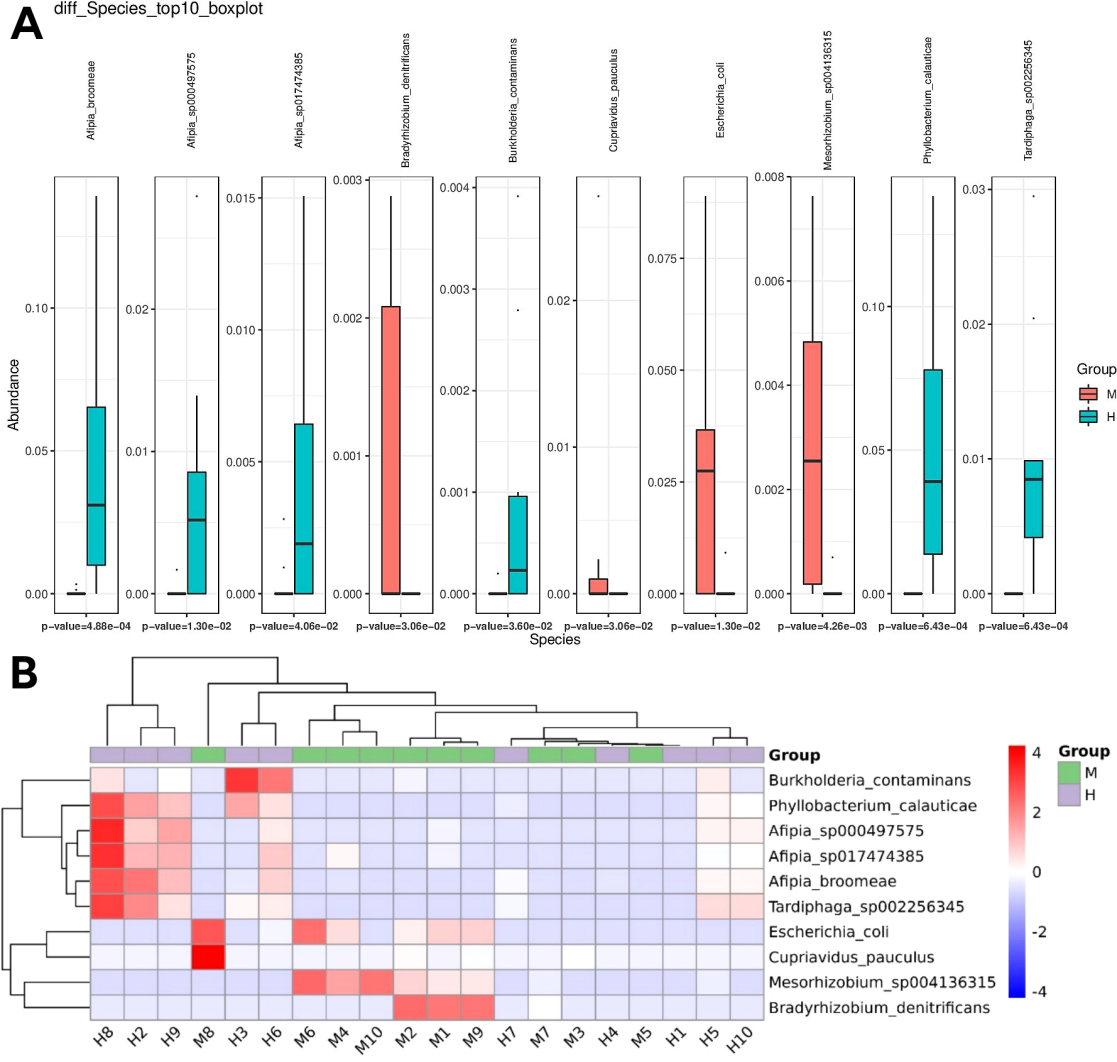
Differential abundance of microbial species between Modic change and herniated disc tissues. **(A,B)** The boxplot **(A)** and heatmap **(B)** show the differences in bacterial species in group H and group M by Wilcoxon test.

We analyzed differences in the microbial composition between group M and the group H at the phylum, genus, and species levels (**Table 2**). There were no significant differences between the two groups at the phylum level. At the genus level, group M had significantly higher abundances of *Escherichia* and significantly lower abundances of *Phyllobacterium, Afipia, Mesorhizobium, Tardiphaga, Brevundimonas*, and *Burkholderia* than those in group H. At the species level, we found that *Escherichia_coli, Cupriavidus_pauculus*, and *Bradyrhizobium_denitrificans* were significantly enriched in group M, while *Afipia_broomeae, Phyllobacterium_calauticae, Tardiphaga_sp002256345, Mesorhizobium_sp004136315, Afipia_sp000497575, Burkholderia_contaminans*, and *Afipia_sp017474385* were more frequently identified in group H (**Figures 4A, B**). We also identified 14 fungal species in IVD tissues; however, there were no significant differences in abundance between the two groups. Detection rates of species with differential abundance between Modic change and herniated disc groups were shown in **Table 3**.

**Table 2:** Microbial composition between Modic change and herniated disc tissues at the genus, and species levels.

Taxa		Average relative abundance (%)		
		Herniated disc	Modic change disc	P-value
Genus	*Phyllobacterium*	4.864	0	0.001
	*Afipia*	5.963	0.103	0.001
	*Mesorhizobium*	0.007	0.293	0.004
	*Tardiphaga*	1.074	0.094	0.009
	*Escherichia*	0.108	2.943	0.027
	*Brevundimonas*	0.346	0.018	0.036
	*Burkholderia*	0.111	0.002	0.036
Species	*Afipia_broomeae*	4.731	0.047	0.001
	*Phyllobacterium_calauticae*	4.855	0	0.001
	*Tardiphaga_sp002256345*	0.964	0	0.001
	*Mesorhizobium_sp004136315*	0.007	0.293	0.004
	*Afipia_sp000497575*	0.677	0.017	0.013
	*Escherichia_coli*	0.092	2.943	0.013
	*Bradyrhizobium_denitrificans*	0	0.086	0.031
	*Cupriavidus_pauculus*	0	0.314	0.031
	*Burkholderia_contaminans*	0.090	0.002	0.036
	*Afipia_sp017474385*	0.383	0.038	0.041

**Table 3:** Detection rates of species with differential abundance between Modic change and herniated disc group.

Species	Modic change disc	Herniated disc
*Afipia_broomeae*	20%	90%
*Afipia_sp000497575*	10%	60%
*Afipia_sp017474385*	20%	60%
*Bradyrhizobium_denitrificans*	40%	0
*Burkholderia_contaminans*	10%	50%
*Cupriavidus_pauculus*	40%	0
*Escherichia_coli*	60%	10%
*Mesorhizobium_sp004136315*	70%	10%
*Phyllobacterium_calauticae*	0	80%
*Tardiphaga_sp002256345*	0	80%

**Figure 5:**
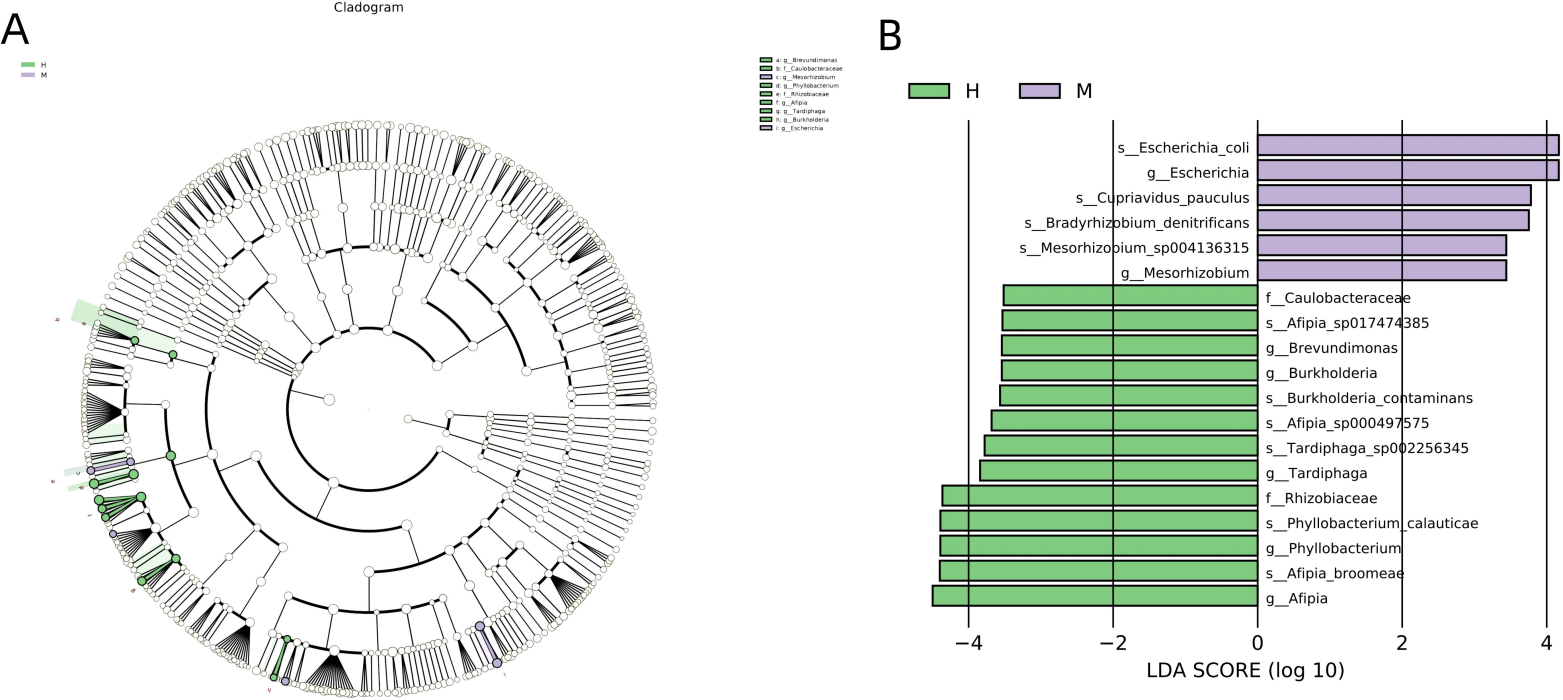
LEfSe was performed to identify differential abundances of bacterial taxa between the two groups. **(A)** The Cladogram represents the taxonomic hierarchical structure biomarkers identified by LEfSe. **(B)** The histogram of LDA score showed significantly different biomarkers between the two groups.

We analyzed the compositions of the two groups using the linear discriminant analysis effect size (LEfSe) method, revealing 19 discriminative features (LDA score ≥ 2.0) with significant differences in relative abundance between group M and group H (**Figures 5A, B**).

### 3.4 Correlations among abundant genera and species

**Figure 6:**
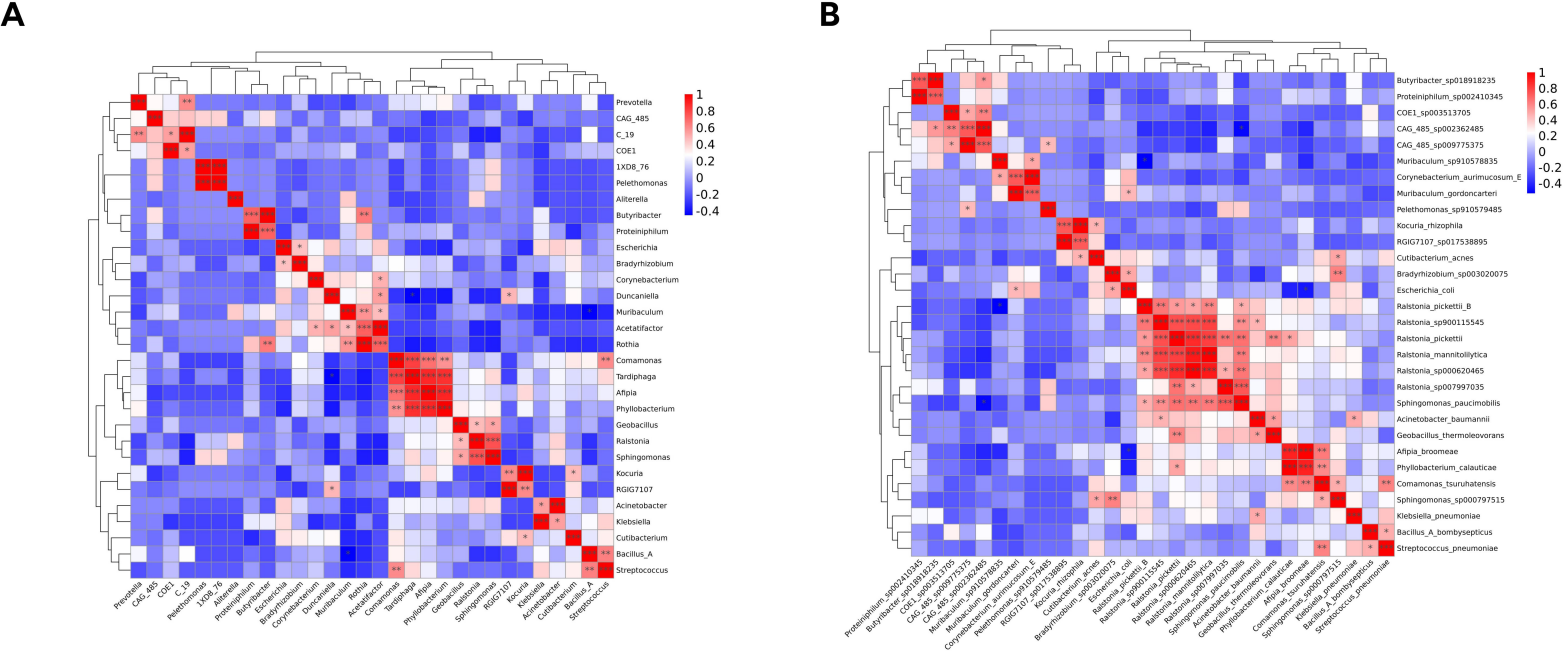
Heatmap of Spearman correlation analysis of the top 30 most abundant genera **(A)** and species **(B)**.

Associations among the top 30 most abundant genera and species were evaluated based on Spearman correlation coefficients. Significant positive and negative correlations were observed among taxa (**Figures 6A, B**). For example, *Streptococcus*, an abundant genus, was positively correlated with several pathogens, such as *Bacillus_A* (*R* = 0.64, P = 0.038) and *Comamonas* (*R* = 0.60, P = 0.038).

### 3.5 Predictive model of group M and group H based on microbiome data

**Figure 7:**
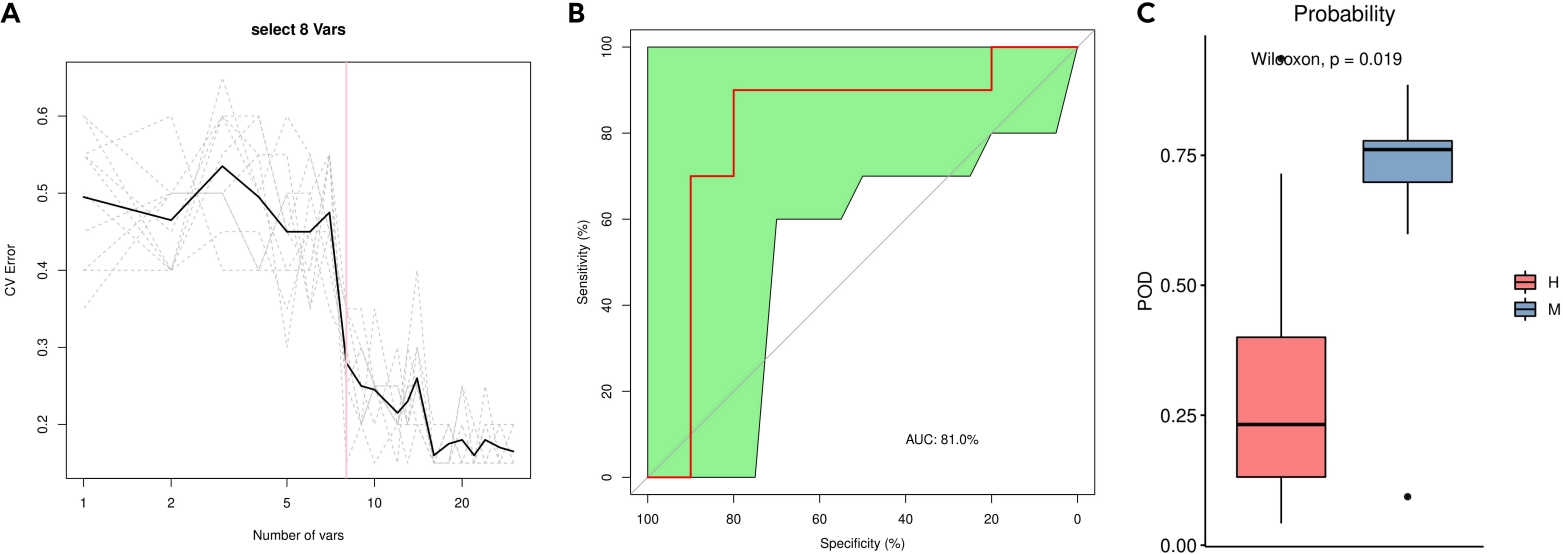
Distinction between group M and group H by random forest analysis. **(A)** Cross-validation error curves showed that 8 species markers were selected as the best set of markers. **(B)** The mean AUC between the two groups reached 81.0%. **(C)** The POD values were significantly higher in the group M than in the group H.

We used a random forest analysis to identify taxa among the top 30 species in relative abundance contributing to differences between groups (**Figure 7**; **Supplementary Figure S1**). Point plots of variable importance revealed that *Afipia_broomeae, Phyllobacterium_calauticae*, and *Escherichia_coli* are the three most important species differing between group M and group H.

We then constructed a random forest classifier based on eight species. The 8-variable random forest model based on the selected bacterial genera shows promising discriminative power for distinguishing between Modic changes and disc herniation The performance of the model was evaluated using cross-validation error curves. The 8-variable model showed the lowest cross-validation error, indicating that it was the optimal set of markers for distinguishing between the two groups. A smaller number of variables helps to reduce the complexity of the model, making it more interpretable and less prone to overfitting. The 8-variable model strikes a balance between sufficient discriminative power and simplicity, which is crucial for clinical applications. The 8 bacterial genera used in the model are: *Phyllobacterium_calauticae, Afipia_broomeae, Escherichia_coli, Comamonas_tsuruhatensis, Ralstonia_pickettii, Bacillus_A_bombysepticus, Ralstonia_pickettii_B* and *Cutibacterium_acnes* (**Supplementary Table S3**). The performance of this optimal marker set was examined by a ROC (receiver operating characteristic) curve analysis, which showed a mean AUC (area under the curve) of 81.0% between group M and group H, indicating good discriminative power. The probability of disease (POD) was estimated as the ratio of the number of randomly generated decision trees that predict samples in group M to that for group H. The POD values were significantly higher for group M than for group H (P = 0.019), indicating that the model based on microbial species markers has a high predictive accuracy for differentiating between Modic changes and herniated discs.

### 3.6 Differences in predicted functions between group M and group H

**Figure 8:**
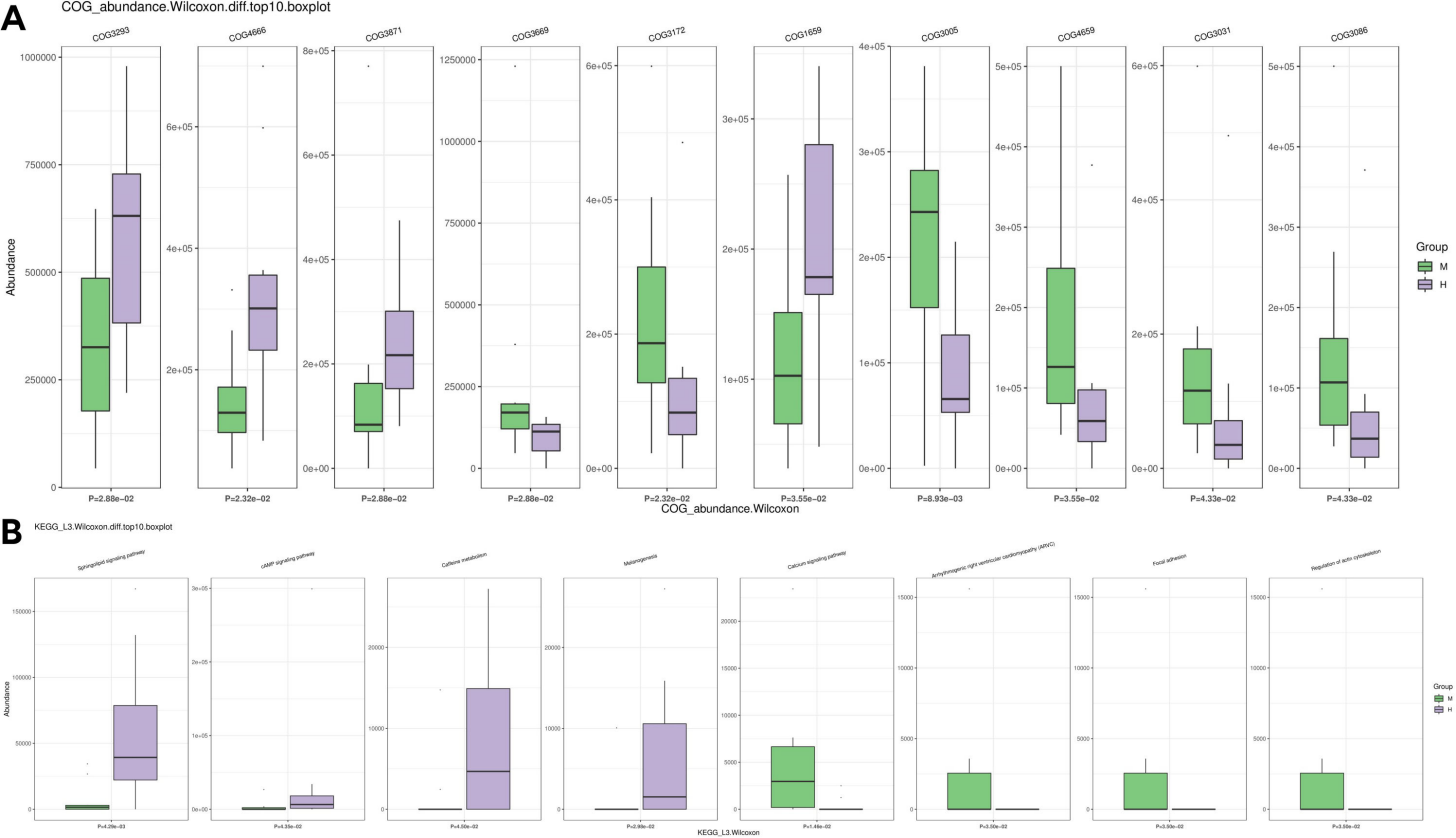
Differences in microbial function between groups by functional annotation analysis. **(A)** Results of COG function prediction for the top 10 most significant differences. **(B)** Results of KEGG function prediction for the top 10 most significant differences.

We further compared the predicted functions of the differentially abundant taxa between the two groups. We identified 4093 COGs that differed between group M and group H; the top five were COG3293 (Transposase), COG4666 (TRAP-type uncharacterized transport system, fused permease components), COG3871 (General stress protein 26 (function unknown)), COG3669 (Alpha-l-fucosidase) and COG3172 (Nicotinamide riboside kinase) (**Figure 8A**). A KEGG pathway enrichment analysis revealed 342 related signaling pathways between group M and group H; the top five were Sphingolipid signaling pathway, cAMP signaling pathway, Caffeine metabolism, Melanogenesis, and Calcium signaling pathway (**Figure 8B**).

## 4 Discussion

Our study is the first to apply 2bRAD-M sequencing to analyze the microbial taxa associated with Modic changes and herniated discs at the species level. Previous studies have identified a range of bacterial genera associated with IVDs, particularly in the context of Modic changes. However, these studies were limited to genus-level identification due to the low microbial biomass in IVD tissues. Our study provides species-level resolution, revealing a more detailed and comprehensive view of the microbial communities in IVDs.

Modic changes in IVDs are associated with several factors, including degenerative processes, inflammation, biomechanical factors, genetic predisposition, infection, metabolic factors, and smoking. Furthermore, microorganisms are likely to influence the occurrence and progression of Modic changes. Microbiota analysis provides a new dimension to the diagnosis of Modic changes by identifying specific microbial taxa that may contribute to the pathogenesis of these conditions. Unlike MRI, which is limited to structural imaging, microbiota analysis can reveal the presence of subclinical infections that may initiate inflammation and degeneration. For example, the enrichment of Escherichia coli in Modic change discs suggests an infective origin, which can be targeted with specific antibiotics. This approach can lead to more accurate diagnosis and tailored treatment plans, potentially reducing the need for invasive procedures. Low back pain has long been linked to infections in the gastrointestinal and urogenital tracts, particularly infections with *Bacillus cereus* and *Citrobacter braakii/freundii* (Rask, 1977; Ford, 1979; Abrutyn et al., 1996). There is a potentially regional association between the natural habitats of these bacteria and spinal discs. It is possible that disc degeneration facilitates the invasion of pathogenic organisms and/or hinders their elimination, leading to a painful inflammatory and immunological response (Hennequin et al., 1996). In a study of 120 patients, Aghazadeh et al. detected the bacterial 16S rDNA gene in 38.3% of disc samples, suggesting that bacterial infection contributes to disc-related pathologies, including Modic changes (Aghazadeh et al., 2017). In a 2008 study, Albert et al. claimed that disc herniation could serve as an entry point for bacteria, leading to Modic changes, edema, and inflammation. Other studies have shown that following a breach of the outer annulus fibrous after herniation of the nucleus pulposus, new capillarization occurs around the extruded nucleus pulposus during a short period of time, leading to inflammation and macrophage aggregation (Albert et al., 2008).

Various approaches have been used to document bacteria in the IVD, including culture-dependent methods and molecular techniques, such as PCR and next-generation sequencing. Human lumbar IVDs, even in asymptomatic subjects, are not sterile and harbor a distinct microbiome (Rajasekaran et al., 2020a). These results have demonstrated the presence of a ‘disc microbiome’ and indicate that dysbiosis is an important factor in disc degeneration. Defense response proteins in discs, age-related increases in inflammatory factors, and the upregulation of the complement system further support the notion that subclinical infection initiates inflammation, ultimately leading to disc degeneration (Rajasekaran et al., 2020b; Rajasekaran et al., 2023a). A significant advancement in this area was made by Sterling et al (Stirling et al. 2001), who detected *Cutibacterium acnes* (*C. acnes*) in disc samples obtained during surgery, a finding supported by numerous subsequent studies (Agarwal et al., 2011; Dudli et al., 2016; Li et al., 2016). Recent metagenome sequencing analyses have challenged conventional views, including the notion that discs are sterile, because they are avascular (Rajasekaran et al., 2020a; Rajasekaran et al., 2023b). A wide range of bacteria have been reported, including *C. acnes*, *Staphylococcus aureus*, *Peptostreptococcus* spp., *Micrococcus*, *Neisseria* spp., *Enterobacter*, *Corynebacterium, Brevibacterium, Rothia dentocariosum Enterococcus faecalis, Streptococcus, Gemmiger, Kocuria, Faecalibacterium*, and *Bacillus* (Granville Smith et al., 2022). Rajasekaran et al. found a similar but altered bacterial diversity in degenerated and herniated samples, prompting the proposal that dysbiosis contributes to the etiology of disc degeneration. They also identified an increased abundance of Proteobacteria and significant reductions in Firmicutes, Actinobacteria, and Bacteroidetes as a potential microbial signature related to the progression of diseased discs. These studies provide evidence for the existence of the gut/skin/spine microbiome axis and reveal various pathogens, in addition to *C. acnes*, in degenerated discs (Rajasekaran et al., 2020a; Shanmuganathan et al., 2022). Variation in microbial taxa could be related to many factors, such as the environment, diet, and lifestyle habits. Rajasekaran et al. employed next-generation sequencing to analyze both healthy and diseased intervertebral discs, revealing a wider spectrum of microbial taxa beyond *C. acnes*. The analysis focused on genus-level identification, emphasizing the presence of *C. acnes* and other genera, but did not provide species-level details or detection rates for individual species. Notably, *C. acnes* was not the most prevalent in all three groups. Interestingly, the Modic group exhibited a higher abundance of opportunistic bacteria, such as *Pseudomonas*, *Sphingomonas*, and *Ochrobactrum*, compared to the non-Modic group. This study highlighted the importance of microbial dysbiosis in disc health but did not explore specific functional pathways or mechanisms. It underscores the necessity to consider a broader range of pathogens beyond *C. acnes*, though it does not specify targets for diagnostics or treatments (Rajasekaran et al., 2023b). By contrast, our study provides valuable insights into the microbial composition of IVD tissues, highlighting the potential role of specific bacterial species in Modic changes and disc herniation. The findings align with previous research on microbial dysbiosis in IVD tissues but offer more detailed species-level information. These results have significant clinical implications for targeted treatments and diagnostic strategies.

Although 16S rRNA sequencing analyses have shown that microbial communities are present in IVD tissues, the resolutions were limited to the genus level. In this study, we characterized the microbiomes associated with Modic changes and herniated discs using 2bRAD-M, a new sequencing technology for precise, low-cost species-level analyses applicable to low biomass samples. The primary aim of our study was to explore bacteria contributing to subclinical infection, providing a basis for the treatment of disc degeneration and thereby potentially shifting it from a condition requiring surgery to a manageable medical issue.

In this study, the microbial species richness in herniated disc tissues was slightly higher than that in the Modic change group. However, there were no significant differences in microbial abundance and diversity between group H and group M, which could be explained by the use of disc tissues from patients with lumbar disc herniation as the control group, because we were unable to collect disc tissues from healthy individuals. With respect to beta diversity, we observed some differences in the microbial composition, particularly in the analysis based on binary Jaccard distances, between the two groups.

Understanding the microbial composition of IVDs and the role of specific species in Modic changes and disc herniation can inform the development of targeted treatment strategies. Positive correlations between microbial taxa suggest that these microbes may work synergistically to contribute to the pathogenesis of Modic changes. For instance, the positive correlation between Streptococcus and Bacillus_A indicates that these bacteria may enhance each other’s pathogenic effects, leading to more severe inflammation and tissue damage. Understanding these positive correlations can help in predicting the behavior of the microbial community. If one pathogen is present, the presence of a positively correlated pathogen may be more likely, indicating a potential for a more complex and severe infection. Negative correlations suggest competitive interactions between microbial taxa. For example, if one species is abundant, the presence of a negatively correlated species may be suppressed. This can be beneficial in a clinical setting, as it may indicate that certain beneficial microbes can outcompete pathogenic ones. Identifying negatively correlated taxa can help in the development of probiotic therapies. For instance, if a particular beneficial microbe is found to negatively correlate with a pathogenic one, introducing the beneficial microbe could potentially reduce the pathogenic load and alleviate symptoms. It is unlikely that a single pathogen causes Modic changes. The IVD harbors diverse microbial communities with complex relationships among taxa, as evidenced by correlation analyses, including both positive and negative or competitive interactions. Moreover, a newly constructed random forest model based on an optimal marker set consisting of eight highly abundant species successfully distinguished between the Modic change and herniated disc groups, with an accuracy of 81.0%. The model demonstrated strong predictive power for identifying Modic changes. Therefore, these specific bacterial taxa may play a significant role in Modic change formation and may serve as diagnostic biomarkers. Microbes associated with Modic changes can also secrete extracellular vesicles; accordingly, liquid biopsy may also serve as a means of early diagnosis. Furthermore, our comparisons of COG annotations and KEGG pathways between groups indicated that there are substantial differences in microbiome functions. More detailed mechanistic studies are necessary to better understand the processes involved.

The clinical relevance of our findings is significant. The identification of specific bacterial species associated with Modic changes and disc herniation suggests that microbial dysbiosis may contribute to the distinct clinical manifestations of these conditions. For example, the presence of *Escherichia_coli* in Modic change discs may indicate an infective origin, implicating several bacteria other than *C. acnes* in these changes. The detection rate shows that *Escherichia_coli* is more frequently detected in Modic change discs (60%) compared to herniated discs (10%). This suggests that *Escherichia_coli* may play a role in the development or progression of Modic changes. Clinically, this could imply that *Escherichia_coli* infections might trigger inflammatory responses leading to Modic changes. Similarly, *Bradyrhizobium denitrificans* and *Cupriavidus pauculus* were detected more frequently in Modic change discs. These species could also be potential pathogens contributing to the pathogenesis of Modic changes. This finding can inform the development of targeted treatment strategies, such as antibiotic therapy for managing Modic changes. For example, antibiotics effective against *Escherichia_coli* could be used to treat Modic changes, potentially reducing the need for surgical interventions. Introducing beneficial microbes that outcompete *Escherichia_coli* could help restore a healthy microbiome in the intervertebral discs.

Our study had several limitations. First, for ethical reasons, we could not collect IVD tissues from healthy individuals as a control group. Thus, we used herniated disc tissues as a control. Second, our sample size was small, which limited comparisons between Modic change subtypes and may have affected the accuracy of the results. The small sample size and limited number of genera raise concerns about overfitting. Future studies should address these limitations by using larger sample sizes, including more genera, and conducting independent validation to ensure the model’s robustness and clinical applicability. In addition, our research was descriptive and we did not directly examine the functions of IVD microbes; therefore, it is difficult to determine the causal relationships between microorganisms and Modic changes. Future studies should focus on mechanistic investigations to better understand the processes involved. Open surgery is currently the most common method for the treatment of Modic changes. However, minimally invasive surgery with fewer complications is also used. Analysis of microbial compositions during the progression of Modic changes may provide assistance for surgical staging.

## 5 Conclusion

In summary, our study provides new insights into the microbial composition of IVDs and highlights the potential role of specific bacterial species in the development and progression of Modic changes. The findings have significant clinical implications, suggesting that microbial dysbiosis may contribute to the distinct clinical manifestations of disc diseases and inform the development of targeted treatment strategies. Further research is needed to fully understand the associations between IVD diseases and the microbiome.

## Data Availability

The datasets presented in this study can be found in online repositories. The names of the repository/repositories and accession number(s) can be found below: Sequence Read Archive (SRA) of the National Center for Biotechnology Information (NCBI) under BioProject accession number PRJNA946904.

## References

[B1] Abrutyn E. Berlin J. Mossey J. Pitsakis P. Levison M. Kaye D. (1996). Does treatment of asymptomatic bacteriuria in older ambulatory women reduce subsequent symptoms of urinary tract infection? J. Am. Geriatr. Soc. 44, 293–295. doi: 10.1111/j.1532-5415.1996.tb00917.x 8600199

[B2] Agarwal V. Golish S. R. Alamin T. F. (2011). Bacteriologic culture of excised intervertebral disc from immunocompetent patients undergoing single level primary lumbar microdiscectomy. J. Spinal. Disord. Tech. 24, 397–400. doi: 10.1097/BSD.0b013e3182019f3a 21150662

[B3] Aghazadeh J. Salehpour F. Ziaeii E. Javanshir N. Samadi A. Sadeghi J.. (2017). Modic changes in the adjacent vertebrae due to disc material infection with Propionibacterium acnes in patients with lumbar disc herniation. Eur. Spine J. 26, 3129–3134. doi: 10.1007/s00586-016-4887-4 27885471

[B4] Albert H. B. Manniche C. Sorensen J. S. Deleuran B. W. (2008). Antibiotic treatment in patients with low-back pain associated with Modic changes Type 1 (bone oedema): a pilot study. Br. J. Sports. Med. 42, 969–973. doi: 10.1136/bjsm.2008.050369 18718972

[B5] Chen Z. Cao P. Zhou Z. Yuan Y. Jiao Y. Zheng Y. (2016). Overview: the role of Propionibacterium acnes in nonpyogenic intervertebral discs. Int. Orthop. 40, 1291–1298. doi: 10.1007/s00264-016-3115-5 26820744

[B6] Dhariwal A. Haugli Bråten L. C. Sturød K. Salvadori G. Bargheet A. Åmdal H.. (2023). Differential response to prolonged amoxicillin treatment: long-term resilience of the microbiome versus long-lasting perturbations in the gut resistome. Gut. Microbes 15, 2157200. doi: 10.1080/19490976.2022.2157200 36576106 PMC9809947

[B7] Dudli S. Liebenberg E. Magnitsky S. Miller S. Demir-Deviren S. Lotz J. C. (2016). Propionibacterium acnes infected intervertebral discs cause vertebral bone marrow lesions consistent with Modic changes. J. Orthop. Res. 34, 1447–1455. doi: 10.1002/jor.23265 27101067

[B8] Ford D. K. (1979). The clinical spectrum of Reiter’s syndrome and similar postenteric arthropathies. Clin. Orthop. Relat. Res. 143, 59–65. 159795

[B9] Granville Smith I. Danckert N. P. Freidin M. B. Wells P. Marchesi J. R. Williams F. M. K. (2022). Evidence for infection in intervertebral disc degeneration: a systematic review. Eur. Spine J. 31, 414–430. doi: 10.1007/s00586-021-07062-1 34862912 PMC8873132

[B10] Hennequin C. Bourée P. Hiesse C. Dupont B. Charpentier B. (1996). Spondylodiskitis due to Candida albicans: report of two patients who were successfully treated with fluconazole and review of the literature. Clin. Infect. Dis. 23, 176–178. doi: 10.1093/clinids/23.1.176 8816150

[B11] James S. L. Abate D. Abate K.H. Abay S.M. Abbafati C. Abbasi N.. (2018). Global, regional, and national incidence, prevalence, and years lived with disability for 354 diseases and injuries for 195 countries and territories, 1990–2017: a systematic analysis for the Global Burden of Disease Study 2017. Lancet 392, 1789–1858. doi: 10.1016/S0140-6736(18)32279-7 30496104 PMC6227754

[B12] Jin Z. Wang D. Zhang H. Liang J. Feng X. Zhao J.. (2020). Incidence trend of five common musculoskeletal disorders from 1990 to 2017 at the global, regional and national level: results from the global burden of disease study 2017. Ann. Rheum. Dis. 79, 1014–1022. doi: 10.1136/annrheumdis-2020-217050 32414807

[B13] Kjaer P. Korsholm L. Bendix T. Sorensen J. S. Leboeuf-Yde C. (2006). Modic changes and their associations with clinical findings. Eur. Spine J. 15, 1312–1319. doi: 10.1007/s00586-006-0185-x 16896838 PMC2438570

[B14] Lam T. Chew D. Zhao H. Zhu P. Zhang L. Dai Y.. (2022). Species-resolved metagenomics of kindergarten microbiomes reveal microbial admixture within sites and potential microbial hazards. Front. Microbiol. 13, 871017. doi: 10.3389/fmicb.2022.871017 35418963 PMC8996153

[B15] Li B. Dong Z. Wu Y. Zeng J. Zheng Q. Xiao B.. (2016). Association between lumbar disc degeneration and propionibacterium acnes infection: clinical research and preliminary exploration of animal experiment. Spine (Phila. Pa. 1976) 41, E764–E769. doi: 10.1097/BRS.0000000000001383 26656049

[B16] Minerbi A. Gonzalez E. Brereton N. J. B. Anjarkouchian A. Dewar K. Fitzcharles M. A.. (2019). Altered microbiome composition in individuals with fibromyalgia. Pain 160, 2589–2602. doi: 10.1097/j.pain.0000000000001640 31219947

[B17] Modic M. T. Masaryk T. J. Ross J. S. Carter J. R. (1988a). Imaging of degenerative disk disease. Radiology 168, 177–186. doi: 10.1148/radiology.168.1.3289089 3289089

[B18] Modic M. T. Steinberg P. M. Ross J. S. Masaryk T. J. Carter J. R. (1988b). Degenerative disk disease: assessment of changes in vertebral body marrow with MR imaging. Radiology 166, 193–199. doi: 10.1148/radiology.166.1.3336678 3336678

[B19] Rajasekaran S. Soundararajan D. C. R. Tangavel C. Muthurajan R. Sri Vijay Anand K. S. MatChado M. S.. (2020a). Human intervertebral discs harbour a unique microbiome and dysbiosis determines health and disease. Eur. Spine J. 29, 1621–1640. doi: 10.1007/s00586-020-06446-z 32409889

[B20] Rajasekaran S. Tangavel C. K S S. V. A. Soundararajan D. C. R. Nayagam S. M. MatChado M. S.. (2020b). Inflammaging determines health and disease in lumbar discs-evidence from differing proteomic signatures of healthy, aging, and degenerating discs. Spine J. 20, 48–59. doi: 10.1016/j.spinee.2019.04.023 31125691

[B21] Rajasekaran S. Tangavel C. Vasudevan G. Easwaran M. Muthurajan R. K S S. V. A.. (2023a). Bacteria in human lumbar discs - subclinical infection or contamination? Metabolomic evidence for colonization, multiplication, and cell-cell cross-talk of bacteria. Spine J. 23, 163–177. doi: 10.1016/j.spinee.2022.05.001 35569807

[B22] Rajasekaran S. Vasudevan G. Easwaran M. Devi Ps N. Anand K S. S. V. Muthurajan R.. (2023b). Are we barking up the wrong tree? Too much emphasis on Cutibacterium acnes and ignoring other pathogens”- a study based on next-generation sequencing of normal and diseased discs. Spine J. 23, 1414–1426. doi: 10.1016/j.spinee.2023.06.396 37369253

[B23] Rask M. R. (1977). Low back pain due to neisseria prostatitis: report of three cases. Clin. Orthop. Relat. Res. 127, 120–122. 144040

[B24] Shanmuganathan R. Tangavel C. K S S. V. A. Muthurajan R. Nayagam S. M. MatChado M. S.. (2022). Comparative metagenomic analysis of human intervertebral disc nucleus pulposus and cartilaginous end plates. Front. Cardiovasc. Med. 9, 927652. doi: 10.3389/fcvm.2022.927652 36247458 PMC9554234

[B25] Shmagel A. Langsetmo L. Demmer R. Knights D. Lane N. E. Ensrud K.. (2019). Gut microbiome associations with chronic musculoskeletal pain in older men. Osteoarthritis. Cartilage. 27, S73. doi: 10.1016/j.joca.2019.02.103

[B26] Stirling A. Worthington T. Rafiq M. Lambert P. A. Elliott T. S. (2001). Association between sciatica and Propionibacterium acnes. Lancet 357, 2024–2025. doi: 10.1016/S0140-6736(00)05109-6 11438138

[B27] Sun Z. Huang S. Zhu P. Tzehau L. Zhao H. Lv J.. (2022). Species-resolved sequencing of low-biomass or degraded microbiomes using 2bRAD-M. Genome Biol. 23, 36. doi: 10.1186/s13059-021-02576-9 35078506 PMC8789378

[B28] Sun Z. Liu J. Zhang M. Wang T. Huang S. Weiss S. T.. (2023). Removal of false positives in metagenomics-based taxonomy profiling via targeting Type IIB restriction sites. Nat. Commun. 14, 5321. doi: 10.1038/s41467-023-41099-8 37658057 PMC10474111

[B29] Sun J. X. Xia Q. D. Zhong X. Y. Liu Z. Wang S. G. (2023). The bladder microbiome of NMIBC and MIBC patients revealed by 2bRAD-M. Front. Cell Infect. Microbiol. 13, 1182322. doi: 10.3389/fcimb.2023.1182322 37351184 PMC10282653

[B30] Wang S. Meyer E. McKay J. K. Matz M. V. (2012). 2b-RAD: a simple and flexible method for genome-wide genotyping. Nat. Methods 9, 808–810. doi: 10.1038/nmeth.2023 22609625

[B31] Zhang Y. H. Zhao C. Q. Jiang L. S. Chen X. D. Dai L. Y. (2008). Modic changes: a systematic review of the literature. Eur. Spine J. 17, 1289–1299. doi: 10.1007/s00586-008-0758-y 18751740 PMC2556462

