# Supplementary material for: 2bRAD-M reveals differences in microbial communities between Modic changes and disc herniation
Source: Front Cell Infect Microbiol. 2025 Mar 21;15:1449873. doi: 10.3389/fcimb.2025.1449873 (PMC11968760; doi:10.3389/fcimb.2025.1449873)
Supplement: Supplementary file 1 [file Table1.docx]

**Table S1. Adaptors and primers used for 2bRAD-M library preparation**

| **Adaptors** | **Sequence (5’ - 3’)** |
| --- | --- |
| Adap-1 sense | ACACTCTTTCCCTACACGACGCTCTTCCGATCTNN |
| Adap-2 sense | GTGACTGGAGTTCAGACGTGTGCTCTTCCGATCTNN |
| Adap antisense | AGATCGGAAGAGC |
| **Primers** | **Sequence (5’ - 3’)** |
| Primer1 | ACACTCTTTCCCTACACGACGCT |
| Primer2 | GTGACTGGAGTTCAGACGTGTGCT |
| 5UDI Primer | AATGATACGGCGACCACCGAGATCTACACXXXXXXXXACACTCTTTCCCTACACGACGCTCTTCCGATCT |
| 7UDI Primer | CAAGCAGAAGACGGCATACGAGATXXXXXXXXGTGACTGGAGTTCAGACGTGTGCTCTTCCGATCT |

**Table S2. Change in data volume during quality control: raw reads, enzyme reads, and clean reads and percentage of clean reads**

| **Sample** | **Raw reads** | **Enzyme reads** | **Clean reads** | **Percent** |
| --- | --- | --- | --- | --- |
| H_1 | 7931117 | 5851346 | 5519950 | 69.60% |
| H_2 | 7112935 | 6498693 | 6143113 | 86.37% |
| H_3 | 6710562 | 5381708 | 5095639 | 75.93% |
| H_4 | 5908648 | 4838220 | 4573270 | 77.40% |
| H_5 | 6174317 | 5253149 | 4963622 | 80.39% |
| H_6 | 6306188 | 5188856 | 4897878 | 77.67% |
| H_7 | 7784421 | 7257603 | 6852094 | 88.02% |
| H_8 | 7511337 | 6235692 | 5897379 | 78.51% |
| H_9 | 7645833 | 6755444 | 6364351 | 83.24% |
| H_10 | 8292012 | 6691829 | 6334368 | 76.39% |
| M_1 | 7150571 | 6596329 | 6223627 | 87.04% |
| M_2 | 22234290 | 20295882 | 19115140 | 85.97% |
| M_3 | 7585850 | 5767141 | 5438642 | 71.69% |
| M_4 | 7775394 | 7088023 | 6704067 | 86.22% |
| M_5 | 7061548 | 5691987 | 5387123 | 76.29% |
| M_6 | 6613061 | 6051767 | 5731440 | 86.67% |
| M_7 | 6717736 | 6360460 | 6012310 | 89.50% |
| M_8 | 6928885 | 4987392 | 4700903 | 67.85% |
| M_9 | 6174137 | 5280789 | 4967728 | 80.46% |
| M_10 | 6703846 | 5156088 | 4856075 | 72.44% |

**Table S3. List of the 8 species in the optimal marker set**

| 1  2  3  4  5  6  7  8 | *Phyllobacterium_calauticae*  *Afipia_broomeae*  *Escherichia_coli*  *Comamonas_tsuruhatensis*  *Ralstonia_pickettii*  *Bacillus_A_bombysepticus*  *Ralstonia_pickettii_B*  *Cutibacterium_acnes* |
| --- | --- |

**
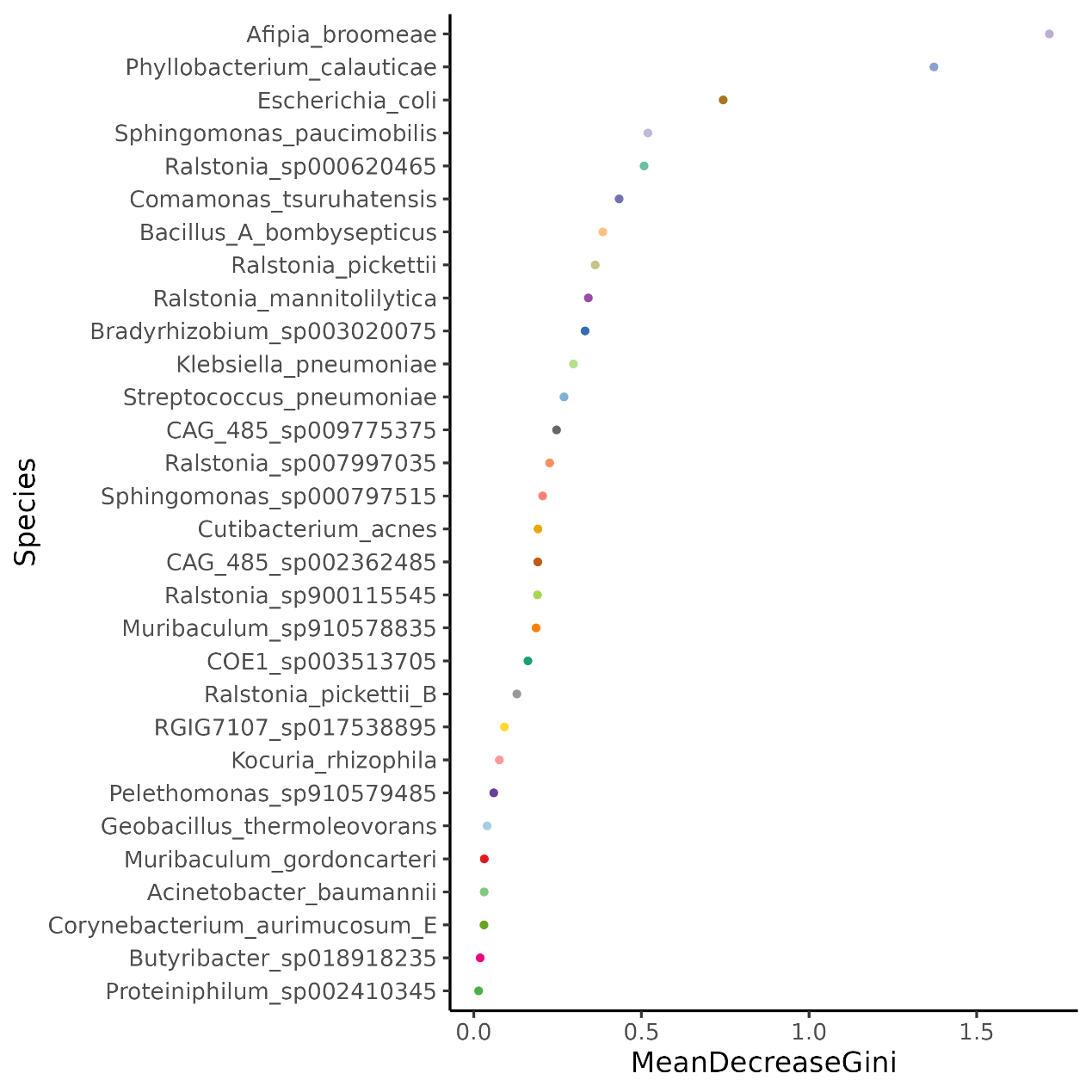
**

**Figure S1. Species importance point diagram. MeanDecreaseGini indicates the importance measure.**
